# Chemical variability in the essential oil of leaves of Araçá (*Psidium guineense* Sw.), with occurrence in the Amazon

**DOI:** 10.1186/s13065-018-0428-z

**Published:** 2018-05-10

**Authors:** Pablo Luis B. Figueiredo, Renan C. Silva, Joyce Kelly R. da Silva, Chieno Suemitsu, Rosa Helena V. Mourão, José Guilherme S. Maia

**Affiliations:** 1 Programa de pós-graduação em Química, Universidade Federal do Pará, 66075-900 Belém, PA Brazil; 2 Faculdade de Química, Universidade Federal do Pará, Belém, PA Brazil; 3 Programa de Pós-Graduação em Biotecnologia, Universidade Federal do Pará, Belém, PA Brazil; 4 Laboratório de Botânica, Universidade Federal do Oeste do Pará, Santarém, PA Brazil; 5 Laboratório de Bioprospecção e Biologia Experimental, Universidade Federal do Oeste do Pará, Santarém, PA Brazil

**Keywords:** *Psidium guineense*, Myrtaceae, essential oil composition, chemical variability

## Abstract

**Background:**

*Psidium guineense*, known as Araçá, is a Brazilian botanical resource with commercial application perspectives, based on the functional elements of its fruits and due to the use of its leaves as an anti-inflammatory and antibacterial agent. The essential oils of leaves of twelve specimens of Araçá were analyzed by GC and GC-MS to identify their volatile constituents and associate them with the biological activities reputed to the plant.

**Results:**

In a total of 157 identified compounds, limonene, α-pinene, β-caryophyllene, *epi*-β-bisabolol, caryophyllene oxide, β-bisabolene, α-copaene, myrcene, muurola-4,10(14)-dien-1-β-ol, β-bisabolol, and *ar*-curcumene were the primary components in descending order up to 5%. Hierarchical Cluster Analysis (HCA) and Principal Component Analysis (PCA) displayed three different groups with the following chemical types: limonene/α-pinene, β-bisabolene/*epi*-β-bisabolol, and β-caryophyllene/caryophyllene oxide. With the previous description of another chemical type rich in spathulenol, it is now understood that at least four different chemotypes for *P. guineense* should occur.

**Conclusions:**

In addition to the use of the Araçá fruits, which are rich in minerals and functional elements, it should be borne in mind that the knowledge of the chemical composition of the essential oils of leaves of their different chemical types may contribute to the selection of varieties with more significant biological activity.

## Background

Myrtaceae comprises 132 genera and 5671 species of trees and shrubs, which are distributed mainly in tropical and subtropical regions of the world, particularly South America, Australia and Tropical Asia [1]. It is one of the most prominent families in Brazil, represented by 23 genera and 1034 species, with occurrence in all regions of the country [2, 3]. *Psidium* is a genus with at least 60 to 100 species, occurring from Mexico and Caribbean to Argentina and Uruguay. Therefore, it is naturally an American genus, although *P. guajava*, *P. guineense* and *P. cattleyanum* are subtropical and tropical species in many other parts of the world [4].

*Psidium guineense* Swartz [syn. *Guajava guineensis* (Sw.) Kuntze, *Myrtus guineensis* (Sw.) Kuntze, *Psidium araca* Raddi, *P. guyanense* Pers., *P. laurifolium* O. Berg, *P. rotundifolium* Standl., *P. sprucei* O. Berg, among others [5] (www.tropicos.org/Name/22102032) is a native shrub or small tree up to about 6 m high occurring in all Brazilian biomes, commonly known as Araçá. It has a berry-type fruit with yellow, red or purple peel and whitish pulp, rich in minerals and functional elements, such as vitamin C and phenolic compounds [6–9]. The leaves and pulp of Araçá have been used as an anti-inflammatory remedy for wound healing and oral antibacterial agent [10, 11], as well as it presented antibacterial activity against pathogenic microorganisms [11–13]. Some essential oils of Araçá were previously described: Foliar oil from a specimen growing in Arizona, USA, with predominance of β-bisabolene, α-pinene and limonene [14]; foliar oil from a specimen collected in Roraima, Brazil, with β-bisabolol, *epi*-α-bisabolol and limonene as the main constituents [15]; and another foliar oil from a specimen sampled in Mato Grosso do Sul Brazil, where spathulenol was the primary volatile compound [16].

The present work aimed at investigating the variability of the chemical composition of the essential oils of different specimens of *Psidium guineense*, occurring in the Amazon region, to contribute to the knowledge of its chemical types.

## Experimental

### Plant material

The leaf samples of twelve *Psidium guineense* specimens were collected in Pará state, Brazil. Collection site and voucher number of each specimen are listed in Table 1. The plant vouchers after the identification were deposited in the Herbaria of Embrapa Amazônia Oriental, in Belém (IAN) and Santarém (HSTM), Pará state, Brazil. The leaves were dried for two days in the natural environment and, then, subjected to essential oil distillation.

**Table 1:** Identification data and collection site of the specimens of *Psidium guineense*

Samples	Collection site	Herbarium Nº	Local coordinates
PG-01	Curuçá, PA, Brazil	IAN-195396	0°72’65” S/47°84’07” W
PG-02	Curuçá, PA, Brazil	IAN-195397	0°43’40” S/47°50’58” W
PG-03	Curuçá, PA, Brazil	IAN-195398	0°72’67” S/47°85’13” W
PG-04	Curuçá, PA, Brazil	IAN-195399	0°72’57” S/47°84’84” W
PG-05	Curuçá, PA, Brazil	IAN-195400	0°72’57” S/47°84’07” W
PG-06	Santarém, PA, Brazil	HSTM-3611	2°27’48.7” S/54°44’04” W
PG-07	Monte Alegre, PA, Brazil	HSTM-6763	1°57’24.9” S/54°07’07.8” W
PG-08	Monte Alegre, PA, Brazil	HSTM-6763	1°57’24.9” S/54°07’07.8” W
PG-09	Santarém, PA, Brazil	HSTM-6775	2°25’14.6” S/54°44’25.8” W
PG-10	Santarém, PA, Brazil	HSTM-3603	2°25’08.4” S/54°44’28.3” W
PG-11	Santarém, PA, Brazil	HSTM-6769	2°29’16.8” S/54°42’07.9” W
PG-12	Ponta de Pedras, PA, Brazil	HSTM-6759	2°31’08.3” S/54°52’25.8” W

### Isolation and analysis of the composition of oils

The leaves were ground and submitted to hydrodistillation using a Clevenger-type apparatus (3 h). The oils were dried over anhydrous sodium sulfate, and their yields were calculated by the plant dry weight. The moisture content of the samples was calculated using an Infrared Moisture Balance for water loss measurement. The procedure was performed in duplicate.

The oils were analyzed on a GCMS-QP2010 Ultra system (Shimadzu Corporation, Tokyo, Japan), equipped with an AOC-20i auto-injector and the GCMS-Solution software containing the NIST (Nist, 2011) and FFNSC 2 (Mondello, 2011) libraries [17, 18]. A Rxi-5ms (30 m x 0.25 mm; 0.25 μm film thickness) silica capillary column (Restek Corporation, Bellefonte, PA, USA) was used. The conditions of analysis were: injector temperature of 250 °C; Oven temperature programming of 60-240 °C (3 °C/min); Helium as carrier gas, adjusted to a linear velocity of 36.5 cm/s (1.0 mL/min); split mode injection for 1 μL of sample (oil 5 μL : hexane 500 μL); split ratio 1:20; ionization by electronic impact at 70 eV; ionization source and transfer line temperatures of 200 and 250 °C, respectively. The mass spectra were obtained by automatic scanning every 0.3 s, with mass fragments in the range of 35-400 m/z. The retention index was calculated for all volatile components using a homologous series of C8-C20 n-alkanes (Sigma-Aldrich, USA), according to the linear equation of Van den Dool and Kratz (1963) [19]. The quantitative data regarding the volatile constituents were obtained by peak-area normalization using a GC 6890 Plus Series, coupled to FID Detector, operated under similar conditions of the GC-MS system. The components of oils were identified by comparing their retention indices and mass spectra (molecular mass and fragmentation pattern) with data stored in the GCMS-Solution system libraries, including the Adams library (2007) [20].

### Statistical analysis

The multivariate analysis was performed using as variables the constituents with content above than 5%. For the multivariate analysis, the data matrix was standardized by subtracting the mean and then dividing it by the standard deviation. For hierarchical cluster analysis, the complete linkage method and the Euclidean distance were used. Minitab software (free 390 version, Minitab Inc., State College, PA, USA), was used for these analyzes.

## Results and discussion

### Yield and composition of the oils

**Fig. 1:**
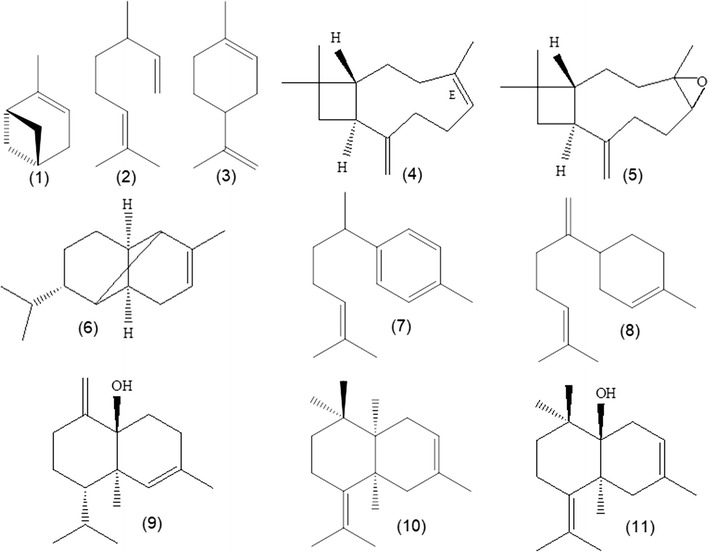
Main constituents identified in the oils of *P. guineense*: (1) α-pinene, (2) myrcene, (3) limonene, (4) β-caryophyllene, (5) caryophyllene oxide, (6) α-copaene, (7) *ar*-curcumene, (8) β-bisabolene, (9) muurola-4,10(14)-dien-1-β-ol, (10) epi-β-bisabolol, (11) β-bisabolol

*Psidium guineense* is a botanical resource that presents commercial application perspectives, based on its fruits and functional elements, as well as due to the use of its leaves as anti-inflammatory and antibacterial agent [6–14]. For this study were selected twelve Araçá specimens, with occurrence in various localities of Pará state (PA), Brazil (see Table 1), and which showed different composition for the leaf oils. The yields of the oils from these twelve Araçá samples ranged from 0.1 to 0.9%, where the higher yields were from specimens sampled in the Northeast of Pará, Brazil (0.4-0.9%), and the lower yields were from plants collected in the West of Pará, Brazil (0.1-0.3%). The identification of the constituents of the oils by GC and GC-MS was 92.5% on average, with a total of 157 compounds, where limonene (0.3-47.4%), α-pinene (0.1-35.6%), β-caryophyllene (0.1-24.0%), *epi*-β-bisabolol (6.5-18.1%), caryophyllene oxide (0.3-14.1%), β-bisabolene (0.1-8.9%), α-copaene (0.3-8.1%), myrcene (0.1-7.3%), muurola-4,10(14)-dien-1-β-ol (1.6-5.8%), β-bisabolol (2.9-5.6%), and *ar*-curcumene (0.1-5.0%) were the primary components, in descending order up to 5% (see Figure 1 and Table 2). In general, the constituents identified in oils belong to the terpenoids class, with the following predominance: monoterpene hydrocarbons (0.9-76.9%), oxygenated sesquiterpenes (5.2-63.5%), sesquiterpene hydrocarbons (5.6-46.7%), and oxygenated monoterpenes (1.9-8.8%).

**Table 2:** Yield and volatile composition of twelve essential oil samples of *P. guineense*

RI_(C)_	RI_(L)_	Constituents (%)	PG-01	PG-02	PG-03	PG-04	PG-05	PG-06	PG-07	PG-08	PG-09	PG-10	PG-11	PG-12
848	846^a^	(2*E*)-Hexenal					0.3	0.1						
850	850^a^	(3*Z*)-Hexenol					0.2	0.1		0.1	0.1			
933	932^a^	*α-Pinene*	*35.6*	*26.1*	*17.7*	*13.4*	*34.0*	*26.4*	2.0	0.8	1.0	1.3	0.1	0.6
946	948^a^	α-Fenchene	0.1		0.1		0.1							
957	952^a^	Benzaldehyde	0.3	0.5	1.1	0.8	0.9	0.6	0.1	0.4	0.3	0.3	0.5	0.1
977	974^a^	β-Pinene	2.1	1.8	1.4	1.3	1.7	3.9	0.1					0.3
985	981^a^	6-methyl-5-Hepten-2-one			0.2		0.1		0.1	0.4	0.1		0.1	0.1
990	988^a^	*Myrcene*	0.2	1.4	1.2	1.4	1.3	1.6	0.1	0.1	0.6	0.7	0.1	*7.3*
1005	1003^a^	*p*-Mentha-1(7),8-diene		0.5	0.9	1.0	0.7	0.3	0.1	0.2	0.7	1.2		0.1
1016	1014^a^	α-Terpinene		0.1		0.1		0.1						
1023	1020^a^	*p*-Cymene	0.3	0.5	1.0	0.7	1.4	0.5	0.2	0.3	0.4	0.3	0.1	0.6
1028	1024^a^	*Limonene*	3.7	*30.7*	*30.4*	*26.5*	*37.2*	*14.0*	4.3	*9.6*	*23.4*	*47.4*	0.3	*5.4*
1031	1032^b^	1,8-Cineole	0.3	0.1	0.1	0.1	0.1	0.1	0.1	0.2	0.1		1.7	0.8
1035	1032^a^	(*Z*)-β-Ocimene	0.1		0.1	0.1			0.1				0.1	0.1
1046	1044^a^	(*E*)-β-Ocimene	0.1		0.2	0.1		0.1	0.8				0.1	0.1
1057	1054^a^	γ-Terpinene	0.6	0.4	0.7	0.6	0.3	0.9			0.2	0.2	0.1	0.1
1088	1086^a^	Terpinolene	0.1	0.1	0.2	0.1	0.1	0.3			0.1			0.1
1100	1095^a^	Linalool	0.1		0.1	0.1		0.1		0.2	0.1		0.1	0.1
1114	1114^a^	*endo*-Fenchol	0.1	0.1	0.1		0.1	0.1						
1116	1113^b^	4,8-dimethyl-(*E*)-Nona-1,3,7-triene	0.1		0.1									
1120	1122^b^	*trans*-*p*-Mentha-2,8-dien-1-ol			0.1	0.1	0.1							
1125	1122^a^	α-Campholenal	0.1		0.1		0.1	0.1						
1130	1131^b^	Limona ketone								1.6				
1134	1133^a^	*cis*-*p*-Mentha-2,8-dien-1-ol					0.1			0.1	0.1			0.1
1138	1136^a^	*trans*-*p*-Menth-2-en-1ol												0.1
1139	1135^a^	*trans*-Pinocarveol	0.4	0.1	0.4	0.1	0.4	0.4	0.2					
1148	1145^a^	Camphene hydrate	0.1	0.1	0.1	0.1	0.1	0.2						
1161	1165^b^	Hydrocinnamaldehyde			0.9	1.5	0.5							
1166	1165^a^	Borneol	0.2	0.1	0.2	0.1	0.2	0.3						
1177	1174^a^	Terpinen-4-ol	0.1	0.1	0.2	0.1	0.2	0.3			0.1			
1186	1187^a^	*trans*-*p*-Mentha-1(7),8-dien-2-ol		0.1				0.1		0.4	0.2			
1187	1189^a^	*trans*-Isocarveol			0.4		0.2							
1191	1186^a^	α-Terpineol	1.0	0.6	1.3	0.4	1.0	1.7	0.2	0.2	0.1		0.1	0.1
1218	1215^a^	*trans*-Carveol			0.2		0.1	0.1		0.1	0.1			
1221	1218^a^	*endo*-Fenchyl acetate	0.7	0.2	0.4	0.3	0.4	0.7						
1226	1227^a^	*cis*-p-Mentha-1(7),8-dien-2-ol			0.4		0.2	0.1		0.3	0.1			
1243	1239^a^	Carvone			0.1					0.1	0.1			
1267	1261^a^	*cis*-Chrysanthenyl acetate		0.1	0.1	0.1	0.1	0.4						0.1
1286	1287^a^	Bornyl acetate	1.5	0.6	0.7	0.5	0.9	1.5	0.1					0.1
1300	1298^a^	*trans-*Pinocarvyl acetate	1.5	0.3	0.3	0.2	0.8	1.6						
1324	1322^a^	Methyl geranate		0.2	0.6	0.6	0.4		0.3	0.3	0.9	2.0	0.3	
1326	1324^a^	Myrtenyl acetate	0.1					0.2						
1336	1335^a^	δ-Elemene	0.2	0.1			0.1	0.1		0.1		2.3		
1338	1339^a^	*trans*-Carvyl acetate			0.1	0.1	0.2	0.1						
1364	1359^a^	Neryl acetate	0.1	0.1	0.1			0.1						
1367	1369^a^	Cyclosativene	0.1		0.1	0.1								
1378	1374^a^	*α-Copaene*	*8.1*	*6.2*	*8.1*	*7.2*	3.0	3.7	4.2	4.7	2.5		1.1	0.3
1383	1379^a^	Geranyl acetate	0.1	1.1	1.0	1.7	0.6	0.8	0.2	0.2	1.9	0.5	0.8	
1401	1401^a^	*iso*-Italicene							0.5	0.6	0.6	0.2	0.1	
1406	1405^a^	Sesquithujene							0.1		0.1		0.1	
1412	1410^a^	α-Cedrene							0.8	0.8	1.0	0.4	0.5	
1416	1407^a^	Acora-3,7(14)-diene							0.9	0.6	1.0	0.5		
1423	1417^a^	*β-Caryophyllene*	*6.1*	2.8	0.1	0.1	0.8	*5.2*	1.4		1.0	0.9	1.1	*24.0*
1426	1419^a^	β-Cedrene							0.1	0.3	0.1		0.1	
1431	1430^a^	β-Copaene							0.2	0.2	0.2		0.1	0.1
1435	1434^a^	γ-Elemene												0.2
1436	1432^a^	*trans*-α-Bergamotene							0.3	0.3	0.3		0.2	
1436	1435^b^	Perillyl acetate	0.1	0.1	0.1	0.2	0.1	0.1			0.2	0.4		
1440	1439^a^	Aromadendrene	0.2	0.1		0.2		0.2	0.2	0.2				
1441	1439^a^	Phenyl ethyl but-2-anoate												0.4
1444	1440^a^	(*Z*)-β-Farnesene							0.2					
1444	1442^a^	Guaia-6,9-diene								0.3				
1447	1445^a^	*epi*-β-Santalene							0.1		0.1		0.1	
1452	1449^a^	Amorpha-4,11-diene							0.3		0.3			
1452	1453^a^	Geranyl acetone			0.1								0.2	
1455	1452^a^	α-Humulene	0.9	0.7	0.3	0.5	0.1	0.9	0.4		0.1		0.2	2.8
1458	1454^a^	(*E*)-β-Farnesene							1.0	0.1	0.5	0.2	0.3	0.1
1460	1457^a^	β-Santalene							1.2		1.1	0.5	0.5	
1461	1460^a^	*allo*-Aromadendrene	0.2	0.2	0.3	0.3	0.1	0.1						
1464	1464^a^	α-Acoradiene							1.3	1.1	1.3	0.6	0.7	
1467	1469^a^	β-Acoradiene							0.4	0.3	0.4	0.2	0.2	
1471	1471^a^	4,5-di-*epi*-Aristolochene				0.1		0.1	0.1	0.1				0.1
1474	1474^a^	10-*epi*-β-Acoradiene							0.4	0.3	0.4		0.2	
1477	1475^a^	γ-Gurjunene		0.3				0.3						
1477	1476^a^	β-Chamigrene												1.0
1479	1478^a^	γ-Muurolene			0.4	0.8	0.1		0.3	0.5	0.2		0.1	
1479	1481^a^	γ-Curcumene							0.4		1.1	0.8	0.7	
1482	1479^a^	*ar* *-Curcumene*							*5.0*	4.6	2.5	0.6	1.6	0.1
1486	1481^a^	γ-Himachalene	1.0	0.9									0.4	
1488	1488^a^	β-Selinene	0.7	0.8	1.0	3.8	0.5	3.2	3.0	3.7	0.1			3.2
1495	1493^a^	α-Zingiberene									0.4	0.3	0.7	
1497	1498^a^	α-Selinene			0.9	3.7	0.3	2.7	4.3	2.4				3.2
1502	1500^a^	α-Muurolene	0.4	0.3	0.5	0.5	0.1	0.2	0.3	0.4	0.2		0.1	0.2
1502	1506^a^	(*Z*)-α-Bisabolene	0.1						0.8	0.3	1.0	0.7	0.6	0.1
1509	1505^a^	(*E*,*E*)-α-Farnesene												2.6
1509	1511^a^	δ-Amorphene				0.4								
1510	1508^b^	*β-Bisabolene*	0.1						*8.9*	4.0	*6.4*	*5.2*	4.0	
1512	1514^a^	β-Curcumene							2.0	0.1	3.6	2.9	2.5	
1516	1513^a^	γ-Cadinene	0.3	0.3	0.3	0.4	0.1	0.2	2.9	0.5				0.2
1516	1514^a^	(*Z*)-γ-Bisabolene							0.9		1.1	1.0	1.0	
1519	1520^a^	7-*epi*-α-Selinene				0.1		0.1						0.1
1522	1524^a^	δ-Cadinene	1.0	1.9	1.7	2.6	0.3	0.7		0.8	2.7	1.9		0.7
1525	1521^a^	β-Sesquiphellandrene											1.8	
1532	1529^a^	(E)-γ-Bisabolene							2.7		2.3	2.0	1.4	0.1
1534	1533^a^	*trans*-Cadina-1,4-diene	0.1	0.1	0.1	0.1		0.1						
1534	1536^a^	Italicene ether							0.2	0.5	0.2		0.5	
1539	1540^a^	10-*epi*-*cis*-Dracunculifoliol							0.1	0.4	0.1			
1543	1540^b^	(*E*)-α-Bisabolene							0.8		0.6	0.4	0.4	
1543	1545^a^	Selina-3,7(11)-diene												0.8
1544	1544^a^	α-Calacorene			0.2	0.3	0.1	0.3		0.7				
1559	1559^a^	Germacrene B						0.1			1.1			0.4
1565	1561^a^	*E*-Nerolidol	0.3	0.1	0.4	0.2		0.1	1.0	1.3		0.9	2.2	0.2
1570	1571^a^	Caryolan-8-ol												0.4
1572	1570^a^	Caryophyllenyl alcohol	0.3					0.2						
1579	1578^b^	*ar*-Tumerol							0.3	0.6	0.1			
1580	1577^a^	Spathulenol	0.7						0.4	0.6				
1584	1590^a^	Globulol		0.1		0.4								
1585	1586^a^	Gleenol			0.3									
1586	1582^a^	*Caryophyllene oxide*	2.5	0.7			0.6	2.7	1.0		0.3		1.2	*14.1*
1589	1590^a^	β-Copaen-4-α-ol			0.5	0.1	0.2	0.3	0.2	0.8				
1594	1592^a^	Viridiflorol	0.2	0.9	0.2	0.1	0.1	0.1	0.2	0.3			0.2	0.3
1596	1595^a^	Cubeban-11-ol							0.1	0.2				
1599	1600^a^	Guaiol												0.5
1601	1600^a^	Cedrol							0.4	0.4	0.5		0.8	
1609	1619^a^	(*Z*)-8-hydroxy-Linalool							0.9	0.7	0.1			
1611	1613^b^	Humulene Epoxide	0.4	0.1	0.1		0.1							1.0
1615	1613^b^	Copaborneol					0.4							
1617	1618^a^	1,10-di-*epi*-Cubenol					0.2							1.7
1625	1622^a^	10-*epi*-γ-Eudesmol							1.3	1.0	1.7	0.7	2.1	
1630	1627^a^	*epi*-Cubenol							1.5	3.4	0.7	0.5		
1631	1632^a^	α-Acorenol							1.5	1.1	1.8	1.2	4.3	
1632	1630^a^	*Muurola-4,10(14)-dien-1-β-ol*	*5.8*	2.4	3.6	2.3	1.6	2.6						
1635	1636^a^	β-Acorenol							0.4	0.5	0.3		0.8	
1637	1636^a^	Gossonorol							1.0	1.6	0.5	0.3	1.1	
1639	1638^a^	Caryophylla-4(12),8(13)-dien-5β-ol	1.3	0.3			0.3	2.1						1.5
1639	1642^b^	Caryophylla -4(12),8(13)-dien-5α-ol	3.1											
1641	1638^a^	*epi*-α-Cadinol	1.9	1.8	1.7	1.7	0.6	1.3	1.1	1.6	0.4	0.8	1.4	
1645	1640^a^	*epi*-α-Murrolol	1.1	0.9					1.2		0.3			2.6
1646	1640^a^	Hinesol							0.6	1.8	0.7	0.4	1.1	
1649	1644^a^	α-Muurolol			1.2	1.1	0.4	0.8			1.1	1.0	1.6	3.1
1653	1649^a^	β-Eudesmol				0.1		0.1	0.2	0.1				0.7
1654	1652^a^	α-Cadinol	1.8	2.0	1.8						0.5	0.4	2.4	
1655	1651^a^	Pogostol							3.8	4.8		0.1		
1659	1658^a^	Selin-11-en-4α-ol				4.2		3.7						4.4
1659	1668^b^	Intermedeol				0.2								0.5
1660	1656^a^	α-Bisabolol Oxide B											2.3	
1671	1670^a^	*epi* *-β-Bisabolol*							*8.1*	*6.5*	*9.5*	*8.2*	*18.1*	
1674	1674^a^	*β-Bisabolol*							2.9	1.9	3.6	3.9	*5.6*	
1675	1671^a^	14-hydroxy-9-*epi*-β-Caryophyllene	1.4					0.7						1.3
1677	1675^a^	Cadalene						0.1		0.6				
1678	1674^a^	Helifolenol A								0.6	0.2			
1680	1679^a^	Khusinol			0.3	0.2								
1685	1683^a^	*epi*-α-Bisabolol							1.0	0.8	1.3	1.2	2.5	
1687	1685^a^	α-Bisabolol							2.8	4.0	2.6	2.2	3.4	
1692	1692^a^	Acorenone												0.2
1696	1696^b^	Juniper camphor												0.8
1698	1700^a^	Eudesm-7(11)-en-4-ol							0.1					0.1
1714	1713^a^	(2*E*,6*Z*)-Farnesal	0.2	1.3	1.5	2.7	0.2				1.0	0.4	2.8	
1721	1722^a^	(2*Z*,6*E*)-Farnesol			3.7	4.6	0.2							0.1
1722	1724^a^	(2*E*,6*E*)-Farnesol	0.4	2.2				1.1		0.2	0.9	0.3	4.9	
1741	1740^a^	(2*E*,6*E*)-Farnesal	0.3	1.9	2.1	3.6	0.4			0.7	1.4	0.6	3.8	
1751	1751^a^	Xanthorrhizol							0.1	0.1				
1757	1753^a^	Isobaeckeol								0.2				
1767	1768^a^	β-Bisabolenal		0.1	0.2	0.2							0.1	
1841	1832^b^	Farnesyl acetate	0.1	0.3	0.2				0.1					
1843	1845^a^	(2*E*,6*E*)-Farnesyl acetate				0.7		0.1	0.1		0.1		0.1	
1962	1958^a^	Geranyl benzoate		0.1	0.1	0.2					0.2		0.1	
Monoterpenes hydrocarbons			42.9	61.6	54.0	45.5	76.9	48.1	7.8	11.0	26.4	51.1	0.9	14.6
Oxygenated monoterpenes			6.6	3.9	7.5	4.7	6.5	8.8	1.9	4.5	3.9	2.8	3.2	1.4
Sesquiterpene hydrocarbons			19.5	14.6	14.0	21.1	5.6	18.6	46.7	28.0	34.3	21.3	20.7	40.1
Oxygenated sesquiterpenes			21.8	15.1	17.8	22.5	5.2	15.9	31.2	36.5	30.2	23.0	63.5	33.6
Others			0.3	1.8	2.1	2.4	1.9	0.8	0.4	0.9	0.5	0.8	0.6	0.8
Total (%)			91.1	97.0	95.4	96.2	96.1	92.2	88.0	80.9	95.3	99.0	88.9	90.5
Yield of oil (%)			0.6	0.6	0.6	0.9	0.4	0.3	0.2	0.1	0.1	0.2	0.2	0.2

Italic: main constituents above 5%

RI_(C)_ retention time calculated; RI_(L)_ retention time of literature

^a^ Adams [20]

^b^ Mondello [18]

Comparing these results with the composition of other essential oils described for the same plant, a specimen of *P. guineense* sampled in Arizona, USA, has also been found to contain β-bisabolene, α-pinene, and limonene as its primary constituents [14]. In addition, the oil from another specimen collected in Roraima, Brazil, presented β-bisabolol as the main component, followed by limonene and *epi*-α-bisabolol [15]. On the other hand, a specimen sampled in Mato Grosso do Sul, Brazil, presented an essential oil with a very high value of spathulenol [16]. Therefore, it is possible that there is a significant variation in the essential oils of different types of Araçá.

### Variability in oils composition

**Fig. 2:**
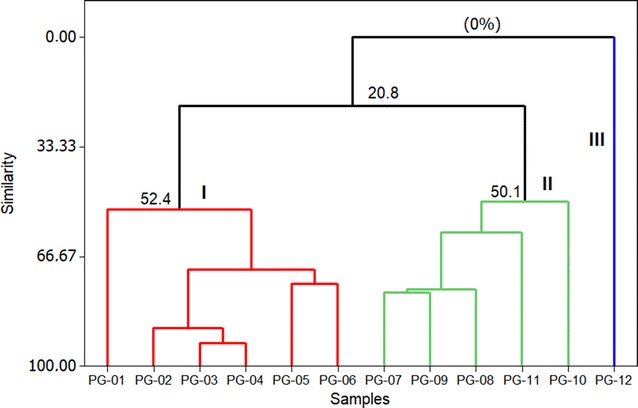
Dendrogram representing the similarity relation in the oils composition of* P. guineense*

**Fig. 3:**
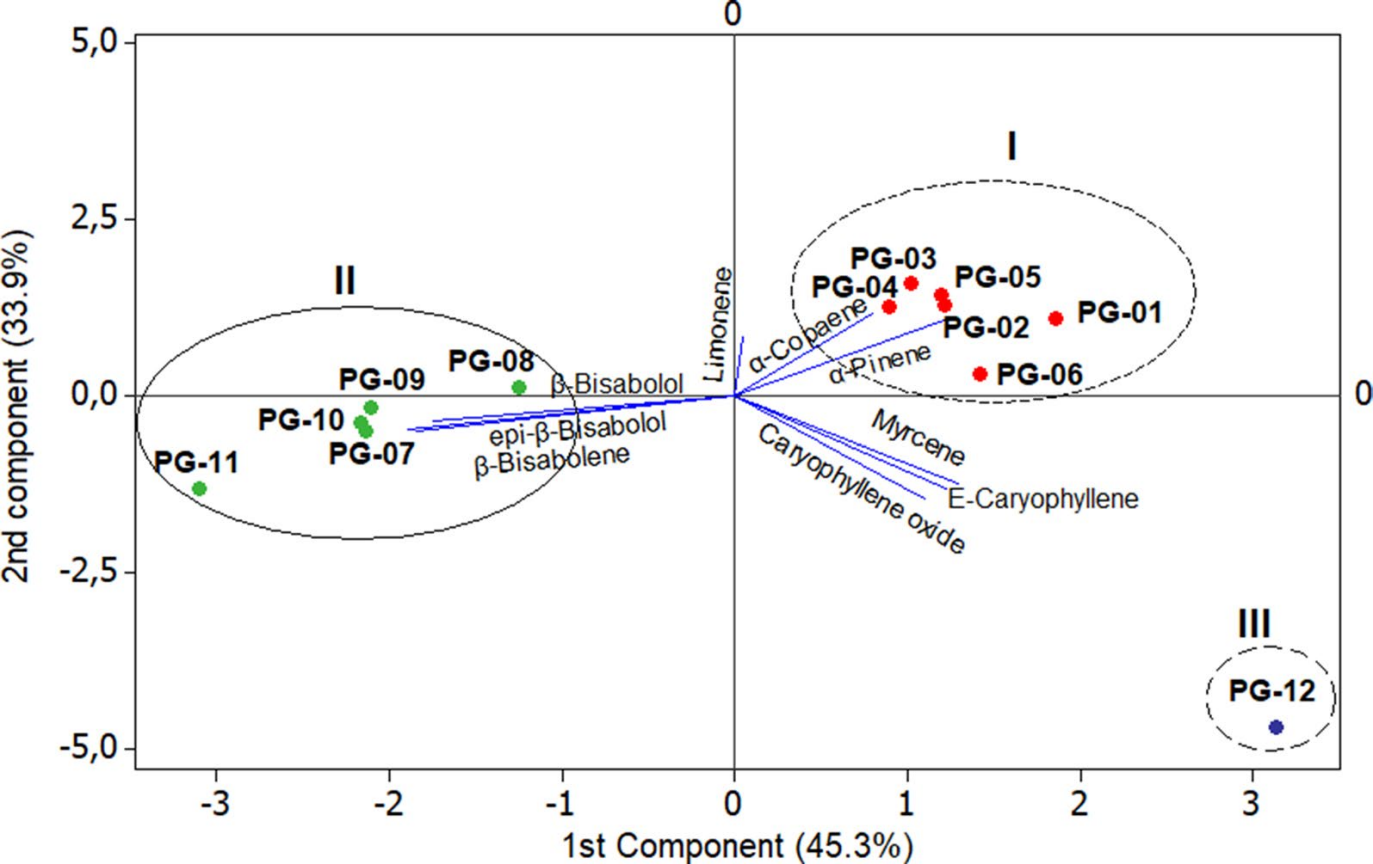
Biplot (PCA) resulting from the analysis of the oils of* P. guineense*

The multivariate analysis of PCA (Principal Component Analysis) (Fig. 2) and HCA (Hierarchical Cluster Analysis) (Fig. 3) were applied to the primary constituents present in oils (content ≥ 5.0%), for the evaluation of chemical variability among the *P. guineense* specimens.

The HCA analysis performed with complete binding and Euclidean distance showed the formation of three different groups. These were confirmed by the PCA analysis, which accounted for 79.5% of the data variance. The three groups were classified as:

*Group I* Characterized by the presence of the monoterpenes α-pinene (13.4-35.6%) and limonene (3,7-37,2%), composed by the specimens PG-01 to PG-06, collected in Curuçá (PG -01 to PG-05) and Santarém (PG-06), Pará state, Brazil, with 49.2% similarity between the samples.

*Group II* Characterized by the presence of the sesquiterpenes β-bisabolene (4.0-8.9%) and epi-β-bisabolol (6.5-18.1%), consisting by PG-07 to PG-10 specimens collected in Monte Alegre (PG-07 and PG-08) and Santarém (PG-09 and PG-10), Pará State, Brazil, with 50.3% similarity between samples.

*Group III* Characterized by the presence of a significant content of β-caryophyllene (24.0%) and caryophyllene oxide (14.1%), constituted by the PG-12 specimen, collected in the city of Ponta de Pedras, Pará state, Brazil, which presented zero% similarity with the other groups.

Thus, based on the study of these essential oils, the multivariate analysis (PCA and HCA) has suggested the existence of three chemical types among the twelve specimens of *P. guineense* collected in different locations of the Brazilian Amazon. It would then be the chemical types α-pinene/limonene (Group I), β-bisabolene/*epi*-β-bisabolol (Group II) and β-caryophyllene/caryophyllene oxide (Group III). Taking into account that two essential oils with a predominance of α-pinene/limonene and β-bisabolene/*epi*-β-bisabolol, respectively, were previously described [14, 15], it is understood that adding these two chemical types to that one rich in β-caryophyllene + caryophyllene oxide, which was a product of this study, besides the other chemical type with a high value of spathulenol, before reported by Nascimento and colleagues (2018) [16], will be now, at least, four chemical types known for the *P. guineense* essential oils.

Several studies have demonstrated the anti-inflammatory activities of limonene, α-pinene and β-caryophyllene, the primary constituents found in the oils of *P. guineense* presented in this paper. Limonene showed significant anti-inflammatory effects both in vivo and in vitro, suggesting a beneficial role as a diet supplement in reducing inflammation [21]; limonene decreased the infiltration of peritoneal exudate leukocytes and reduced the number of polymorphonuclear leukocytes, in the induced peritonitis [22]. α-Pinene presented anti-inflammatory effects in human chondrocytes, exhibiting potential anti-osteoarthritic activity [23], and in mouse peritoneal macrophages induced by lipopolysaccharides [24], being, therefore, a potential source for the pharmaceutical industry. The anti-arthritic and the in vivo anti-inflammatory activities of β-caryophyllene was evaluated by molecular imaging [25].

## Conclusion

In addition to the great use of the fruits of *P. guineense*, which are rich in minerals and functional elements, it is understood that the knowledge of the chemical composition of the essential oils of leaves of their different chemical types may contribute to the selection of varieties with more significant biological activity. The study intended to address this gap.

## Abbreviations

HCA: Hierarchical Cluster Analysis
PCA: Principal Component Analysis
GC: Gas chromatography
GC-MS: Gas chromatography-Mass spectrometry
IAN: Herbarium of Embrapa Amazônia Oriental
HSTM: Herbarium of Santarém
